# Influence of Reagents on the Synthesis Process and Shape of Silver Nanoparticles

**DOI:** 10.3390/ma15196829

**Published:** 2022-10-01

**Authors:** Oksana Velgosova, Lívia Mačák, Elena Čižmárová, Vladimír Mára

**Affiliations:** 1 Institute of Materials and Quality Engineering, Faculty of Materials Metallurgy and Recycling, Technical University of Košice, Letná 9/A, 042 00 Košice, Slovakia; 2 Department of Materials Engineering, Faculty of Mechanical Engineering, Czech Technical University in Prague, Karlovo nám. 13, 121 32 Prague 2, Czech Republic

**Keywords:** silver, nanoparticles, chemical reduction, TEM, nanoparticles shape

## Abstract

The aim of this study was to prepare the silver nanoparticles (AgNPs) via chemical reduction and analyze the impact of used reduction agents: sodium borohydride (NaBH_4_), trisodium citrate (TSC), polyvinylpyrrolidone (PVP), and hydrogen peroxide (H_2_O_2_) on the reduction rate of Ag^+^ ions to Ag^0^, and on nanoparticles shape. It was proven that combinations of reduction agents dramatically influence the synthesis rate of AgNPs and the color of solutions, which depends on the shape and size of nanoparticles. NaBH_4_, TSC, and PVP showed good reduction power. In particular, TSC proved to be a key factor influencing the shape of AgNPs. The shape of nanoparticles influences the color of colloidal solutions. Yellow solutions, where UV-vis absorbance maxima (ABS_max_) are in the wavelength interval 380–420 nm, contain spherical particles with a mean size of 25 nm, whereas the blue shift of ABS_max_ to wavelengths higher than 750 nm indicate the presence of triangular nanoparticles (size interval 18–150 nm). A mixture of spherical, triangular, irregular, and hexagonal nanoparticles give different color, e.g., green. The formation and stability of AgNPs was tracked by UV-vis spectroscopy, size and shape by TEM techniques, and particle size distribution was studied by particle size analyzer.

## 1. Introduction

Nanoparticles are defined as a particle of size 1–100 nm. The oldest known example, where the nanoparticles were used/produced is the Lycurgus Cup from ancient Rome. The glass cup contains gold and silver nano-powders, which are able to change color, the so-called Dichroic effect. The paradox is that, despite the current technical achievements, it is not clear how they made the cups with such optical properties. Silver has accompanied humanity for centuries, and in recent decades, due to current technical progress, its use has been even significantly expanded. The development of science and technology made it possible to produce silver on the nanoscale purposefully. With the reduction of the size of silver particles, unique properties appeared, which silver on the macroscale does not offer. Nano size considerably changed optical, physical, chemical, electrical, thermal, and biological properties due to their surface-to-volume ratio. Thanks to them, nanosilver finds application in various areas of science and technology, e.g., surface enhancer Raman spectroscopy, sensors, and AgNPs are also widely used in medicine as antimicrobial agents, biomedical device coatings, drug delivery carriers, imaging probes, and diagnostic and optoelectronic platforms since they have discrete physical and optical properties [1,2,3,4]. The effect of concentration, shape, and size of the nanoparticles on antibacterial properties has also been documented [5,6]. For instance, Martınez-Castanon et al. studied the effect of shape and size of the nanoparticles on anti-bacterial properties and proved that size has a significant impact [7].

There are several ways to prepare nanoparticles, where physical methods generally offer the production of powdered nanoparticles, while nanoparticles prepared by chemical methods are in the form of colloidal solutions. A very interesting and ecologically acceptable method of preparation is a biological method, the so-called green method, in which biological materials (plants, fungi, algae, etc.) are used as reducing and stabilizing agents [8]. Each of these methods has its advantages and disadvantages, but only chemical reduction allows the synthesis of nanoparticles of different shapes and sizes by simply changing the type and the proportions of the reactants. Another advantage of chemical reduction is the number of available reactants, such as NaBH_4_, hydrazine, ascorbic acid, triethanolamine, glucose, PVA, PVP, cellulose, etc. Metraux and Mirkin, for instance, applied the chemical reduction to prepare Ag-NPs using a mixture of AgNO_3_/NaBH_4_/PVP/TSC/H_2_O_2_ in an aqueous solution at room temperature, and the results revealed the problem with obtaining small sized nanoparticles [9]. Their results and the results of other authors showed that the concentration of the solutions, their amount, order of adding, process conditions, and reducing agent ratios have a fundamental influence on the formation of nanoparticles.

In general, all AgNPs according to their shape are possible to assign to three types: nanorods, “platonic solids”, and triangular prisms. However, in real colloids, mixtures of particles with varying degrees of tip truncation and rounding can be observed. If a significant degree of truncation and rounding occurs such nanoparticles are described as spherical or near spherical. Therefore, if there is a requirement for a certain shape (triangular, rod, etc.) of nanoparticles, ensuring the stability of synthesized nanoparticles is very important [10,11].

The size of the nanoparticles is a very important factor because it affects a lot of parameters of the material, e.g., melting temperature (the smaller the nanoparticles, the lower the melting point), optical properties, toxicity (smaller nanoparticles are more toxic to cells), etc. Depending on the shape, AgNPs have exceptional plasmonic features in both visible and IR regions and have shown significant SERS signals. The shape of the nanoparticles has also a dramatic effect on the absorption and perceived color of the colloidal solution, for example, solutions of spherical nanoparticles are in shades of yellow, triangular in shades of blue and/or purple, and a mixture of different shapes can give green color [12,13]. Silver nanoparticles are also known for the possibility to show a dichroic effect. This phenomenon depends on the presence of highly polymorphic stable nanoparticles or a mixture of differently shaped nanoparticles. Much effort has been made to understand the dichroic phenomenon, and many hypotheses have been proposed to explain the formation of such highly anisotropic structures [14,15,16]. However, the synthesis of such shaped AgNPs and ensuring their stability represents a relatively complicated process.

Several authors describe the process of nanoparticle synthesis [17,18] or analyze the influence of ambient conditions such as the pH of solutions, LED irradiation [19], process temperature, and stirring on AgNPs formation [20,21,22]. However, there are still a lot of questions to solve. Research carried out in the last decade has clearly shown that the optical, catalytic, and other properties of silver nanoparticles are strongly influenced by their shape, size, and size distribution. These parameters are varied by varying the reducing agents and stabilizers. Hence, there is need to understand the influence of reaction agents on the synthesis process.

This work focuses on observing and analyzing the impact of four reactants (NaBH_4_, TSC, PVP, and H_2_O_2_) and their combinations on the synthesis process of silver nanoparticles. Herein, we demonstrate the impact of each of the reactants on the AgNPs synthesis rate, and the resulting shape, size, and stability of the nanoparticles. Controlling the shape and size of the particles, such as spheres, polyhedrons, and prisms, allows the control of plasmon resonances through the entire visible and near IR spectrum, which extends practical applications of nanoparticles.

## 2. Materials and Methods

### 2.1. Materials

Silver nitrate (>98%) purchased from Mikrochem Ltd., Pezinok, Slovakia was used as a silver precursor. Sodium citrate Na_3_C_6_H_5_O_7_ (TSC) (≥99%), polyvinylpyrrolidone (C_6_H_9_NO)_n_ (PVP), hydrogen peroxide H_2_O_2_ (30%), and sodium borohydride NaBH_4_ (≥98%), were also purchased from Mikrochem Ltd., Pezinok, Slovakia, and used as received. De-ionized water was used for preparing all solutions.

### 2.2. Synthesis of AgNPs

The procedure of AgNPs colloids preparation was as follows:

1. AgNO_3_ stock solution with a concentration of 0.11 mM was prepared;
2. TSC, PVP, hydrogen peroxide, and NaBH_4_ solutions with concentrations of 30 mM, 2% *w*/*w*, 30% *w*/*w*, and 100 mM, respectively, were prepared;
3. 86 mL of AgNO_3_ stock solution was poured into each of the fourteen (A-N) Erlenmeyer flasks. Subsequently, 6.68 mL of TSC, 6.68 mL of PVP, 0.24 mL of H_2_O_2_, and 0.4 mL of NaBH_4_ were added in various combinations according to Table 1;
4. the reagents were added in order: AgNO_3_, TSC, PVP, H_2_O_2_, NaBH_4_;
5. prepared solutions were not stirred and were left at the ambient conditions to observe the process of AgNPs synthesis.

### 2.3. Methods

Prepared solutions were analyzed by UV-vis Spectrometer (UNICAM UV-vis Spectrophotometer UV4) on the day 0 (three hours after the preparing) and on the 7th day. The size and morphology of the nanoparticles were studied by means of TEM (JEOL model JEM-2000FX microscope operated at an accelerating voltage of 200 kV). The image analysis (ImageJ software, National Institutes of Health and the Laboratory for Optical and Computational Instrumentation (LOCI, University of Wisconsin LicensePublic Domain, BSD-2 Version 1.53t)) was used for the analysis of Ag nanoparticles’ size distribution.

## 3. Results

Silver nanoparticles (AgNPs) were synthesized by the chemical method using TSC, PVP, H_2_O_2_, and NaBH_4_, Table 1. A lot of reactions take place during the synthesis process, and each one has an important role [14,23,24]. Understanding the roles of reactants is not a simple task, but using various combinations of reactants might help; see Table 1.

**Figure 1:**
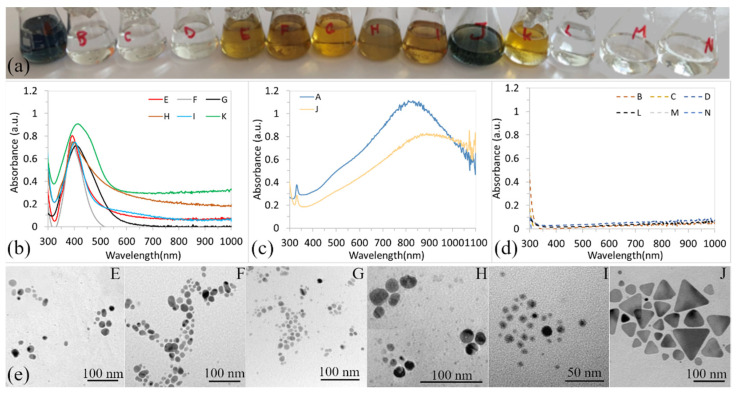
The AgNPs colloids of different colors (**a**); UV-vis of the first group of solution (yellow—E, F, G, H, I, K) (**b**); the second group (blue—A, J) (**c**); the third group—solutions without the nanoparticles (**d**); and the TEM images of AgNPs of colloidal solutions E, F, G, H, I and J (from **left** to **right**) (**e**).

After preparing the solutions (according to Table 1), some of them immediately changed color (A, E, F, G, H, I, J, K), as shown in Figure 1a, which means that silver nanoparticles were formed, but some remained unchanged (B, C, D, L, M, N). Figure 1a shows the change of solution colors (3 h after preparation). According to the color, it is possible to divide the solutions into three groups: the solutions in shades of yellow belong to the first one, the second contains blue-colored solutions, and the third contains transparent, pure solutions. Based on the colors of the solutions, we can suggest that the different shapes or sizes of nanoparticles, if any, were synthesized.

Figure 1b–d shows UV-vis spectra (surface plasmon resonance bands—SPR bands) of prepared solutions. SPR band of the solutions of the first group (E, F, G, H, I, and K) have a very similar shape and their wavelength of ABS_max_ are in the near interval (from 392 to 414 nm), Figure 1b. The same plasmonic properties of solutions indicate nanoparticles of similar shapes and sizes [13].

It is well known that spherical silver nanoparticles with sizes up to 20 nm in diameter have the ABS_max_ around 350–400 nm. Therefore, it is possible to conclude that AgNPs in the colloidal solutions of the first group are spherical and the mean particle diameter should be up to 20 nm. The SPR bands of the second group are shown in Figure 1c. These colloids show two peaks, one sharp peak at 330 nm and a second strong peak at a wavelength higher than 750 nm. Both SPR bands (solutions A and J) are very similar in shape. Based on the results of other authors, the presence of two peaks, with one strong peak at a high wavelength (higher than 510 nm) and a small one at 300 nm indicates the presence of triangular structures of nano prisms [14,19,23,24,25]. Figure 1d shows the SPR bands of solutions B, C, D, L, M, and N. It is clear that the reagents (TSC, PVP, H_2_O_2_, and their combinations) did not have the ability to reduce silver ions, or their reducing ability is very weak.

Figure 1e shows the TEM images of AgNPs synthesized in solutions E, F, G, H, I, and J. All nanoparticles of the first group are spherical, and uniform, their size interval is 1–45 nm (85% of them are up to 25 nm). Nanoparticles of the second groups (A, and J) are mostly triangular with the height of the triangle 18–150 nm, but there are also a few spherical particles with a diameter of 2–30 nm and truncated triangles.

**Figure 2:**
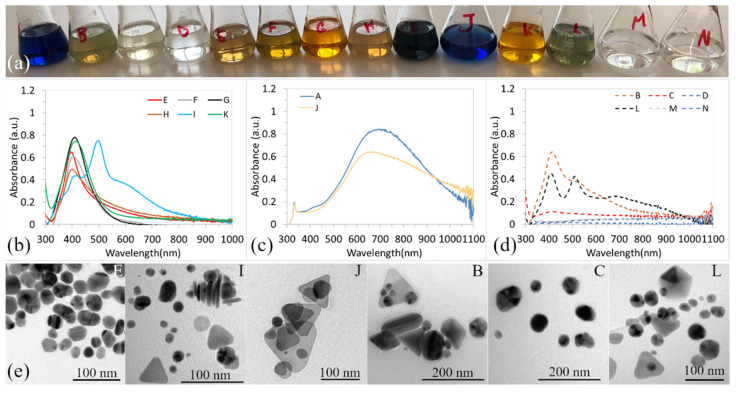
The AgNPs coloids of different color on 7th day of experiment (**a**); UV-vis of the first group of solution (yelow—E, F, G, H, I, K) (**b**); the second group (blue—A, J) (**c**); the third group (**d**) and the TEM images of AgNPs of colloidal solutions E, I, J, B, C and L (from **left** to **right**) (**e**).

After a week, it is possible to observe changes not only in the color of the solutions but also in the shape of the UV-vis spectra. Significant changes were observed in solutions B, C, I, and L. Solution B turned yellow with a shade of green, solution C become a light yellow, I greenish-brown and solution L turned green, Figure 2a. The other solutions of the first group (E, F, G, H, and K) did not change in color dramatically, as shown in Figure 2a, which indicates the good stability of nanoparticles. UV-vis spectra of the yellow solutions are shown in Figure 2b.

One strong and slim peak in solutions E, F, G, and H was observed, Figure 2b, which indicates stabile, spherical, uniform, and small nanoparticles. Figure 2e shows the TEM image of AgNPs synthesized in solution E, where the spherical and uniform nanoparticles of the average size of 20 nm were confirmed. The SPR band of solution I changed dramatically. Moreover, three peaks are evident, as shown in Figure 2b, one peak (more like a shoulder) at a high wavelength of ~600 nm, the second strong one at ~500 nm, and the third peak at 420 nm. The presence of three peaks indicates the presence of nanoparticles with different, irregular shapes, which was also confirmed by TEM, as shown in Figure 2e. The SPR bands of A and J solutions showed a blue shift but strong peaks are still on the area of wavelength >600 nm and the position of the second peak at 330 nm did not change at all, as shown in Figure 2c. TEM confirmed the unchanged, triangular shape in solution J, as shown in Figure 2e, and A (data not shown).

The significant changes can be observed in SPR bands of solutions B, C, and L. Solution C showed a small peak and the change to light yellow, which is typical for solutions of the first group. Small, spherical nanoparticles were observed by TEM, as shown in Figure 2e, in this solution. B and L solutions showed the most significant changes in color, and also in the SPR bands shape, Figure 2d. The strong peak at 415 nm and the second at 520 nm were observed in both solutions. Moreover, solution L has the third broad peak at ~685 nm, indicating the presence of nanoparticles with different shapes, which was confirmed by TEM, as shown in Figure 2e.

**Figure 3:**
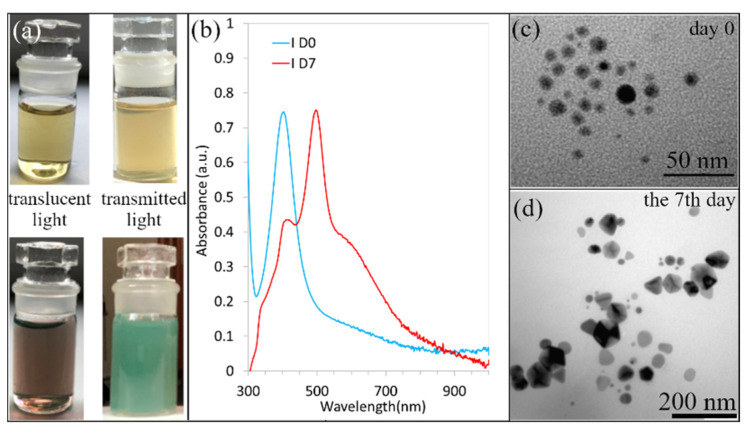
The AgNPs coloids solution I on day 0 (yellow solutions) and 7th day (brown and green solutions) (**a**); UV-vis of the solution I on day 0 and 7th day (**b**); and the TEM images of AgNPs of colloidal solution I on day 0 (**c**) and on the 7th day (**d**).

A strong dichroic effect was observed in samples B, I, and L. Figure 3 shows data for sample I. A noticeable dichroic effect for sample I was observed on the 7th day. Figure 3a shows the color of the solution on day 0, it is clear that the solution was yellow in both the transmitted light and reflected light. On the 7th day, the solutions’ color depended on the light source location. In the transmitted light, it was brown, and green in the reflected light.

It is well known that the color of the solution depends on the shape of the nanoparticles, and this is reflected in the shape of the SPR bands. Figure 3b shows the changes in the UV-vis absorbance spectra of sample I in time. One peak on day 0 (yellow solution) confirmed the presence of spherical nanoparticles, and 3 peaks on the 7th day (brown solution) indicate the mix of differently shaped nanoparticles. TEM images, Figure 3c,d, confirmed the presence of spherical nanoparticles on day 0, and their transformation into a mix of nanoprisms, nanodecahedrons, and near-spherical nanoparticles (the 7th day). This transformation and the presence of different shapes and sizes of nanoparticles caused the formation of the dichroic effect. Moreover, the ratio between the individual shapes is important. We assume that the ratio determines the color of the solution.

## 4. Discussion

As the reaction conditions strongly influence the shape and stability of the nanoparticles, many authors have studied the influence of various factors on the AgNPs synthesis process [13,20,22,25,26,27]. It must be said that the synthesis process is very sensitive to changes in the amount of reducing agents [26,27]. Among reagents mostly used for AgNPs synthesis (TSC, PVP, H_2_O_2_, and NaBH_4_), NaBH_4_ is considered a very strong reducing agent which can reduce most metal salts to elemental metals. The chemical reaction for the reduction of AgNO_3_ in the presence of NaBH_4_ is as follows [26]:

2AgNO_3_ + 2NaBH_4_ → 2Ag^0^ + B_2_H_6_ + 2NaNO_3_ + H_2_↑ (1)

Our results showed that NaBH_4_ adding (solution E) caused an immediate reduction of Ag^+^ ions. The yellow solution formed, and spherical nanoparticles with an average size of 11.8 nm were observed, as shown in Figure 1e. No changes in color or in nanoparticles’ shape/size were observed even after a week. Mirzaei et al. showed that when sodium borohydride was used for the reduction of silver, the reducing reaction was very intense, and they supposed that in the absence of a protective dispersing agent (stabilizer), the resulting particle size would increase as a result of agglomeration effects. Therefore, they used PVP as a surfactant to prevent the growth of AgNPs. We did not observe the agglomeration (solution E) as, in our case, stable near-spherical nanoparticles formed. The growth and agglomeration of nanoparticles were not observed even after a month (data not shown). In the solution, where both PVP and NaBH_4_ were used (solution G), spherical nanoparticles of the average size of 10.5 nm with near size distribution interval were formed, and their size and shape did also not change with time. It follows that PVP, in the presence of NaBH_4_, just insignificantly affects the size of the nanoparticles. We also found that PVP itself is a very weak reducing agent because the nanoparticles in solution C formed on the 7th day. Solution C turned slightly yellow on the 7th day, and UV-vis showed only a very low ABS_max_ value (0.11), as shown in Figure 2b, compared to solutions E and G (ABS_max_ was 0.79 and 0.81, respectively).

Rashid et al. and Pinero et al. prepared AgNPs colloids by TSC and/or NaBH_4_ and showed that both reactants can reduce Ag^+^ ions to Ag^0^ (in both cases yellow solutions were prepared—spherical nanoparticles). Their UV-vis confirmed the presence of AgNPs and they determined the TSC as an agent, which causes a slower growth process than NaBH_4_ [18,21]. Ho et al. prepared AgNPs by TSC, and as in previous mentioned works, the light-yellow colloid solution was prepared, and spherical nanoparticles (35 nm) were confirmed by UV-vis and TEM analysis [17]. These results are in good agreement with our results regarding the reduction process of Ag^+^ ions to Ag^0^, but we found some differences in the effects of both reaction agents.

Based on our results, it can be concluded that TSC, PVP, and NaBH_4_ have reducing ability, which is confirmed by solutions B, C, and E. NaBH_4_ is a strong reducing agent (AgNPs synthesized immediately) and a good stabilizer, TSC and PVP are weak reducing agents (AgNP synthesis was delayed—growth process slower). The mechanism of Ag^+^ cation reactions with TSC and PVP can be expressed by chemical equations:

4Ag^+^ + Na_3_C_6_H_5_O_7_ + 2H_2_O → 4Ag^0^ + C_6_H_5_O_7_H_3_ + 3Na^+^ + H^+^ + O_2_↑ (2)

2Ag^+^ + (C_6_H_9_NO)_n_ → 2Ag^0^ + C_6_H_6_O_6_ + 2H^+^ (3)

Our experiments also showed that PVP (solution C), NaBH_4_ (solution E), and a combination of NaBH_4_ + TSC, NaBH_4_ + PVP, NaBH_4_ + H_2_O_2_, and NaBH_4_ + PVP + H_2_O_2_ (solutions F, G, H, and K) lead to the synthesis of spherical nanoparticles. It is clear that, in all solutions where NaBH_4_ is present, the stable spherical nanoparticles (solutions F, G, H, and K) formed, and synthesis was conducted almost immediately after adding reagents. It is well-known that strong reducing agents have high nucleation ability and favor the formation of many small nanoparticles. Gontijo et al. prepared AgNPs solution by a combination of NaBH_4_ + TSC, and concluded, that the nucleation of nanoparticles occurred by means of an inducing process starting from a supersaturated solution forming AgNPs cores [20]. From the core formed, the nanoparticles were grown by a diffusion process. They also concluded that the colloidal stability of nanoparticles can be explained by the good adsorption of ligands on the surface of colloidal particles [20,28]. Gontijo et al. report that TSC provides surface ligands [20]. Based on our results, we assume that PVP has played a similar role in the synthesis process. since NaBH_4_ is a strong reducing agent that ensures the formation of many nuclei. The synthesis, in solutions where NaBH_4_ was used (C, E–K solutions), results in the formation of small, stable, and spherical nanoparticles. All solutions with such nanoparticles are yellow and ABS_max_ is in interval 384–418 nm, Figure 1a,b and Figure 2a,b. Also, Torres et al. and Van Dong et al. confirmed the uniform spherical and stabile nanoparticles prepared by a combination of PVP + NaBH_4_ [29,30].

On the other hand, H_2_O_2_ as an oxidant has no reducing ability at all (solution D), the presence of H_2_O_2_ even prevents the reduction effect of TSC and PVP (solutions M and N). However, with the combination of TSC + PVP + H_2_O_2_ + NaBH_4_ and TSC + H_2_O_2_ + NaBH_4_ (solutions J and A), the triangular nanoparticles formed immediately after mixing, shown Figure 1c, and remain stable, Table 2. It is obvious that the triangular shape did not depend on PVP. The key combination of reagents for the formation of triangular nanoparticles is TSC, H_2_O_2_, and NaBH_4_.

At similar reaction conditions (presence of TSC + PVP + H_2_O_2_ + NaBH_4_), triangular nanoparticles with good stability were prepared also by Torres et al. and Van Dong et al. [29,30]. On their UV-vis spectra, the triangular shapes were reflected by three different frequency resonances, as shown on the UV-vis spectra of our solution A. The H_2_O_2_ cannot reduce Ag^+^ cations but, based on the latest research [14], it can affect the shape of already formed nanoparticles. According to findings in works [24,31,32], the impact of H_2_O_2_ on silver ions strongly depends on the pH of the solution, H_2_O_2_ can act as a reducing agent under alkaline conditions. Under certain conditions, H_2_O_2_ is also able to change the shape of already synthesized nanoparticles by etching, and truncated triangles are formed [14,23,33]. Less stable, newly formed silver nanoparticles are etched by hydrogen peroxide, establishing an equilibrium between sodium borohydride reduction and hydrogen peroxide oxidation of silver [34]:

2Ag^0^ + 2H_2_O + 2H^+^ → 2Ag^+^ + H_2_O (4)

UV-vis curve showed three, two, and three peaks, respectively. In addition, we found that TSC (solution B), a combination of TSC + PVP (solution L) and TSC + PVP + NaBH_4_ (solution I), leads to the formation of colloids with nanoparticles of various, irregular shapes. Such colloids show a dichroic effect, which has not been observed with any other combination of reagents, as shown in Table 2. Such a phenomenon was observed also by other authors [35,36,37]. Caseri analyzed several works from which, it is clear, that there are discrepancies in the explanation of the dichroic effect and there are several hypotheses about what this effect (in the case of colloidal solutions) causes [36]. Most authors agree that anisotropic metal particles are responsible for the dichroic effect. We suppose that TSC is the first shape determining factor. The combination of TSC with other reactants led to the formation of different shapes of nanoparticles as triangular, spherical, irregular, etc. Moreover, the ratio between differently shaped nanoparticles is important because it determines the position and intensity of absorption peaks, and this is reflected in the color of the colloidal solution.

## 5. Conclusions

The aim of the work was to analyze the impact of different reagents on AgNPs’ synthesis rate, nanoparticles’ size, shape, and their changes in time. Our experiments showed that reacting conditions and relative quantities of reagents must be carefully controlled. Experiments showed that NaBH_4_ is not the one reactant that can reduce silver ions, as TSC and PVP have reduction power, but NaBH_4_ is the fastest. TSC secured the reduction as well as stabilization of nanoparticles. It can be concluded that TSC is a very important reactant in the process of nanoparticle shape influencing. It can be also concluded that TSC is the only factor influencing the synthesis of dichroic nanoparticles. H_2_O_2_ is not a reducing agent, but it is the shape influencing agent (triangular, truncated triangular shape). The size of spherical nanoparticles was in intervals of 1–45 nm and triangular nanoparticles 15–150 nm. The changes in shapes with time were observed in some solutions and even the dichroic effect was observed at certain reagent combinations.

This work has shed light on the key reagents that determine the formation of Ag nanoparticles and ensure their stability. It also showed the roles of each reagent, and provided a repeatable manual for the synthesis of nanoparticles of different shapes, therefore representing a significant step toward understanding the mechanism of AgNPs synthesis by chemical reduction.

## Figures and Tables

**Table 1:** Combination of reagents.

Labeling of Solutions	Reagents: AgNO_3_+	Labeling of Solutions	Reagents: AgNO_3_+
A	TSC + PVP + H_2_O_2_ + NaBH_4_	H	H_2_O_2_ + NaBH_4_
B	TSC	I	TSC + PVP + NaBH_4_
C	PVP	J	TSC + H_2_O_2_ + NaBH_4_
D	H_2_O_2_	K	PVP + H_2_O_2_ + NaBH_4_
E	NaBH_4_	L	TSC + PVP
F	TSC + NaBH_4_	M	TSC + H_2_O_2_
G	PVP + NaBH_4_	N	PVP + H_2_O_2_

**Table 2:** Summary of experimental results for solutions with TSC.

Labeling of Solutions	Reagents: AgNO_3_+	0 Day		7th Day		Solution Colour
		Shape	UV-vis	Shape	UV-vis	
B	TSC	-	-	irregular	2 peaks	dichroic
L	TSC + PVP	-	-	irregular	3 peaks	dichroic
F	TSC + NaBH_4_	● *	1 peak	●	1 peak	yellow
I	TSC + PVP + NaBH_4_	●	1 peak	irregular	3 peaks	dichroic
A	TSC + PVP + H_2_O_2_ + NaBH_4_	▲ ^+^	3 peaks	▲	3 peaks	blue
J	TSC + H_2_O_2_ + NaBH_4_	▲	3 peaks	▲	3 peaks	blue

* spherical shape; ^+^ triangular shape.

## Data Availability

Not applicable.
